# Interactive Web-Based Visualization of Multidimensional Physical and Astronomical Data

**DOI:** 10.3389/fdata.2021.626998

**Published:** 2021-06-24

**Authors:** Faruk Diblen, Luc Hendriks, Bob Stienen, Sascha Caron, Rena Bakhshi, Jisk Attema

**Affiliations:** ^1^ Netherlands eScience Center, Amsterdam, Netherlands; ^2^ High Energy Physics, IMAPP, Radboud University Nijmegen, Nijmegen, Netherlands; ^3^ Nikhef, Amsterdam, Netherlands

**Keywords:** visualization, multidimensional data, machine learning, simulations, particle physics

## Abstract

In this article, we propose expanding the use of scientific repositories such as Zenodo and HEP data, in particular, to better study multiparametric solutions of physical models. The implementation of interactive web-based visualizations enables quick and convenient reanalysis and comparisons of phenomenological data. To illustrate our point of view, we present some examples and demos for dark matter models, supersymmetry exclusions, and LHC simulations.

## 1 Introduction

Practically, any research done in modern physics nowadays is based on (simulated) data. Some examples include the investigation of the Higgs boson properties, the search and exclusion of new models for physics beyond the Standard Model at the LHC, the investigation of gravitational waves, or the identification of dark matter. In all these scientific efforts, the exploration of data with the help of physical models plays a key role. The models are often complex; i.e., they depend on various physical parameters and their interpretation may depend on systematic effects described with the help of additional nuisance parameters.

Traditionally, scientific data are provided by the experiments mainly in the form of one-dimensional histograms and data analysis typically required a comparison of the model to the histogram of data. The scientific models investigated were also of low complexity. Predictions of models that describe physics are typically compared to the data using often time-consuming simulations of the underlying physical processes for a large number of model parameter sets. Finally, the best-fit contours of the models are presented in the form of likelihoods or posterior distributions as functions of a model parameter θ, typically in the form of one- to two-dimensional figures in scientific publications.

In the field of searches beyond the Standard Model, typically 95% confidence level upper limits are provided; i.e., model parameter sets are classified between “excluded” and “allowed.”

**FIGURE 1:**
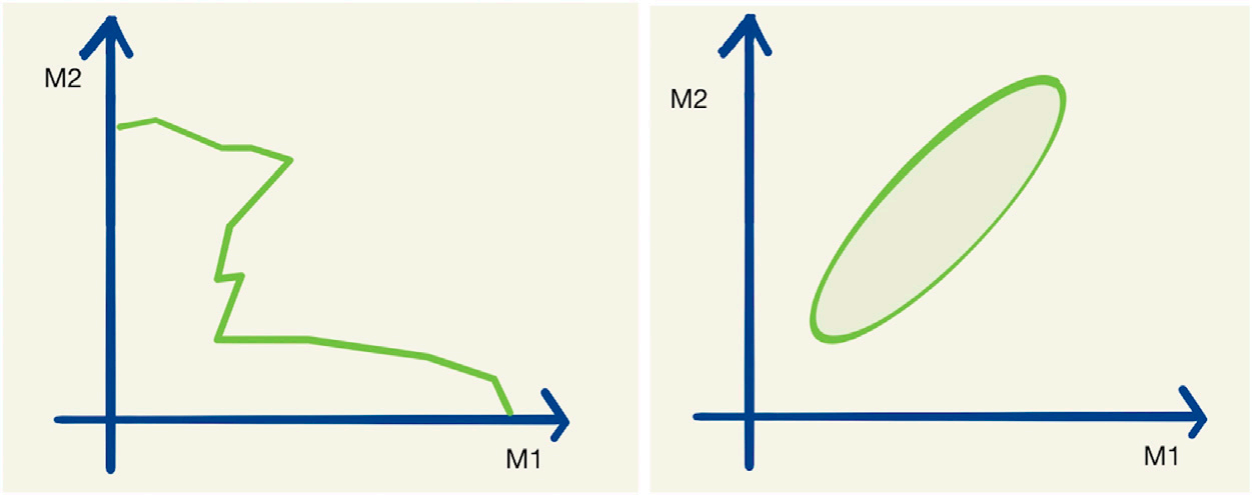
A typical exclusion limit curve **(left)** and a typical maximum likelihood interval **(right)** as a function of the two parameters M1 and M2. Let us assume that the hypothetical real physical model has 20 parameters M1–M20.

In order to visualize high-dimensional data, one can use one- or two-dimensional projections (e.g., some parameter variables have been marginalized or set to best-fit values) or slices of the full space, but this comes at the cost of information contained in the visualization itself. An example of such a one-dimensional exclusion contour and a maximum likelihood interval is given in Figure 1.

The problem becomes even more apparent in the combination and comparison of the results of two different research datasets. To actually visualize data and make a comparison between two datasets, expert knowledge on the creation of those sets is often needed.

There are exciting visualization tools that solve these problems, but these are not open source (Ahlberg, 1996; Heer et al., 2008; Lachev and Price, 2018), require technical expertise, or are generic tools (Metabase, 2020; Superset, 2020). Moreover, open-source packages HEPData-explore (Maguire et al., 2017) and ROOT (Brun and Rademakers, 1997) and *Python* package Matplotlib (Hunter, 2007) are the most popular in the High-Energy Physics community. However, the plots that these tools produce are not interactive. Besides, ROOT, Matplotlib, and another popular *Python* package Plotly. (2020) require programming expertise.

An open and user-friendly visualization tool would also allow researchers with limited time (and limited technical skills) to find new correlations in published data. We believe that such a web-based and easy-to-use data-visualization tool can generate new ideas and accelerate science.

Repositories like Zenodo (European Organization For Nuclear Research and OpenAIRE, 2013) allow us to store likelihood or exclusion boundaries for thousands to millions of different parameter sets. Zenodo is today also widely used to publicly store the results of simulations used in scientific publications.

This article proposes building two additions to this new way of multivariate data explorations and shows working demonstrations.

• **Interactive visualization of data samples:** We believe that there should also be an easy way to visualize, explore, and compare multidimensional data. This allows quick and intuitive visualization and analysis of the model data. We propose a tool for “online” 1–3 dimensional visualization and creating histograms of high-dimensional datasets which could be connected to online repositories such as Zenodo.

• **Generalization of data samples:** As described in Brooijmans et al. (2020), the regression and classification with Machine Learning (ML) allow practical interpolations *in between* the provided model solutions. ML-based interpolations are the best practice also for high-dimensional models. Such tools are built by the community (or could be automatically built on some of the Zenodo datasets). This would allow us to determine exclusion, likelihood, posteriors, etc. for an arbitrary set of model parameters.

In the following, we discuss a few example cases and provide links to working demos built on SPOT (Diblen et al., 2018; Attema et al., 2020) with datasets available on Zenodo via phenoMLdata.org.

## 2 Technical Details of the Software and the Workflow

The primary goal of SPOT is to provide an interactive data exploration environment for high-dimensional datasets. Moreover, SPOT aims to facilitate open science, data sharing, and reuse.

The technical details of SPOT are given in detail in Diblen et al. (2018). In this section, we will give an overview of the software components and explain the workflow.

SPOT consists of three components: the SPOT framework, a front-end, and the SPOT server. The SPOT framework provides classes for datasets, data views, partitions, aggregation, and filtering. The front-end is a web-based application that allows users to upload data, configure dataset properties, and interact with the data. The dashboard of the front-end provides interaction. Using the front-end users can download or share the dashboard provenance files which we will refer to as “session file” in this paper. Both the server and the front-end are written in JavaScript.

**FIGURE 2:**
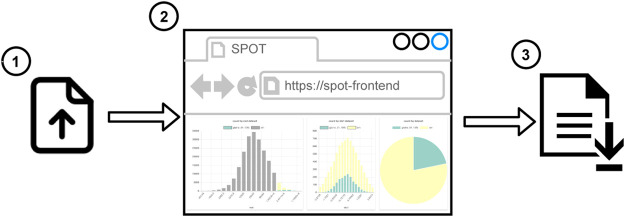
Workflow to use SPOT in client mode.

**FIGURE 3:**
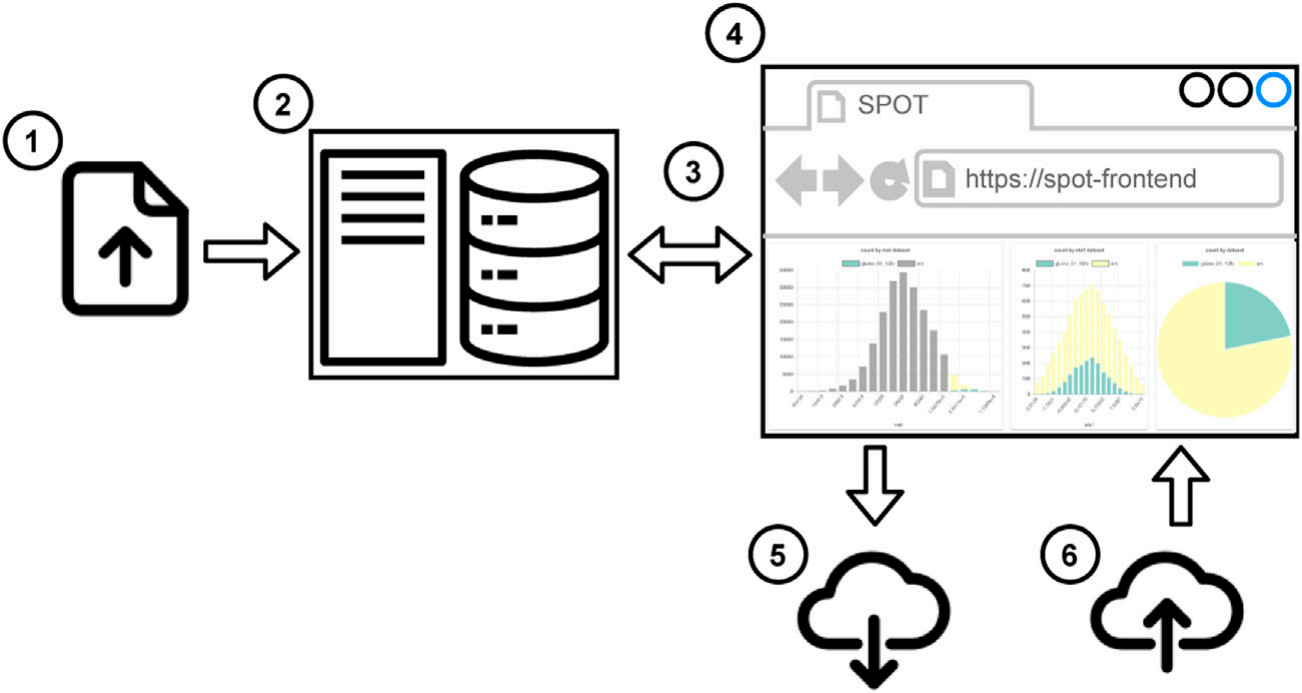
Workflow to use SPOT in server mode.

There are two workflows to use SPOT. In the first one (see Figure 2), the user uploads the local dataset or the session file to the web application. 1) The supported data formats are CSV and JSON. Then the user can start to work on a dashboard. 2) The data partitioning, filtering, and aggregations run in the web browser, so users do not need any additional software. It works even without an Internet connection. When the user completes the analysis, the session file is shared so that another user can reuse or alter the analysis. 3) The second workflow (see Figure 3) is more suitable for big datasets. First, the datasets are uploaded 1) to the SPOT server which uses a PostgreSQL database for storage 2). When the user interacts with the dashboard, SQL queries are sent to the server 3) and the dashboard view is updated 4). Finally, the session file can be saved to share the analysis 5). This session can be uploaded to the front-end to do further analysis using the datasets on the server 6). Both workflows are suitable for internal use such as local network which may be required due to privacy reasons.

The examples demonstrated in this paper were generated using server workflow. The session files were uploaded to Zenodo so that dashboards can be regenerated by the readers. To be fully transparent, the datasets in our database are also added to Zenodo so that readers can also access the raw data. The links and DOIs of the datasets and the session files are provided.

## 3 Demonstration of Interactive Visualization

*phenoMLdata* is an online interactive plotting interface to a database of publicly available datasets, based on the open-source SPOT framework. A prototype of the website can be found at http://spot.phenomldata.org.

The tool allows for the creation of histograms, line plots, pie charts, and scatter plots (both two-dimensional and three-dimensional) of any (combination) of the variables without downloading the data to the user’s computer. As conventional with plotting tools, the properties of these plots, like ranges of the axes, colors, and points sizes can be customized and made data dependent.

During the creation of a plot, all available data from the datasets are plotted. SPOT adds interactivity between the plots by linking them. In a session with a histogram and a scatter plot users can, for example, select a region in the scatter plot. The histogram then automatically alters itself to only show data contained in the user’s selection, making it possible to filter data in real time.

Another advantage is the possibility to compare datasets. The online interface provides access to a database containing the datasets. Any dataset in this database can be selected for visualization. By selecting multiple datasets, it is possible to plot (the same) variables of different datasets in the same plot. This makes it possible to compare, e.g., exclusion boundaries of different papers, projected onto any available plane.

Any visualization made with the tool can be exported as a provenance file (active session) for sharing. Any other user that uploads this file can then use the visualizations for themselves and has access to the full interactive arsenal of SPOT in doing so. This allows us, for example, to interactively explore and validate data and to design data selections. The next section showcases this explicitly: any of the provided examples come with a URL to the provenance file for that specific example. Uploading this file to SPOT opens the full interactive version of the said example.

## 4 Examples

For each example, we provide the “session URL” in the figure captions. All the session files can be found on https://doi.org/10.5281/zenodo.4247860.

### 4.1 Example 1: SUSY-AI

A primary goal of particle physics is to find signals that are predicted by extensions of the Standard Model. These extensions always come with several new parameters, such as the masses and couplings of new particles. The ATLAS experiment provides in ATLAS collaboration. (2015) for a 19-dimensional model of new physics (the so-called pMSSM) and about 310,000 realizations of this model (with randomly selected parameter sets) the information whether this parameter set is excluded (or not) by ATLAS measurements. We want to emphasize that such data are of great use. Online visualizations could encourage (traditionally conservative) experimental collaborations to continue providing high-dimensional data.

These 310,000 model configurations with binary exclusions allowed the construction of an ML classifier to predict the exclusion contour in the full 19-dimensional model space (see Caron et al., 2017). The investigation of the exclusion of the 19-dimensional model parameters is, nevertheless, typically still done through two-dimensional projections.

**FIGURE 4:**
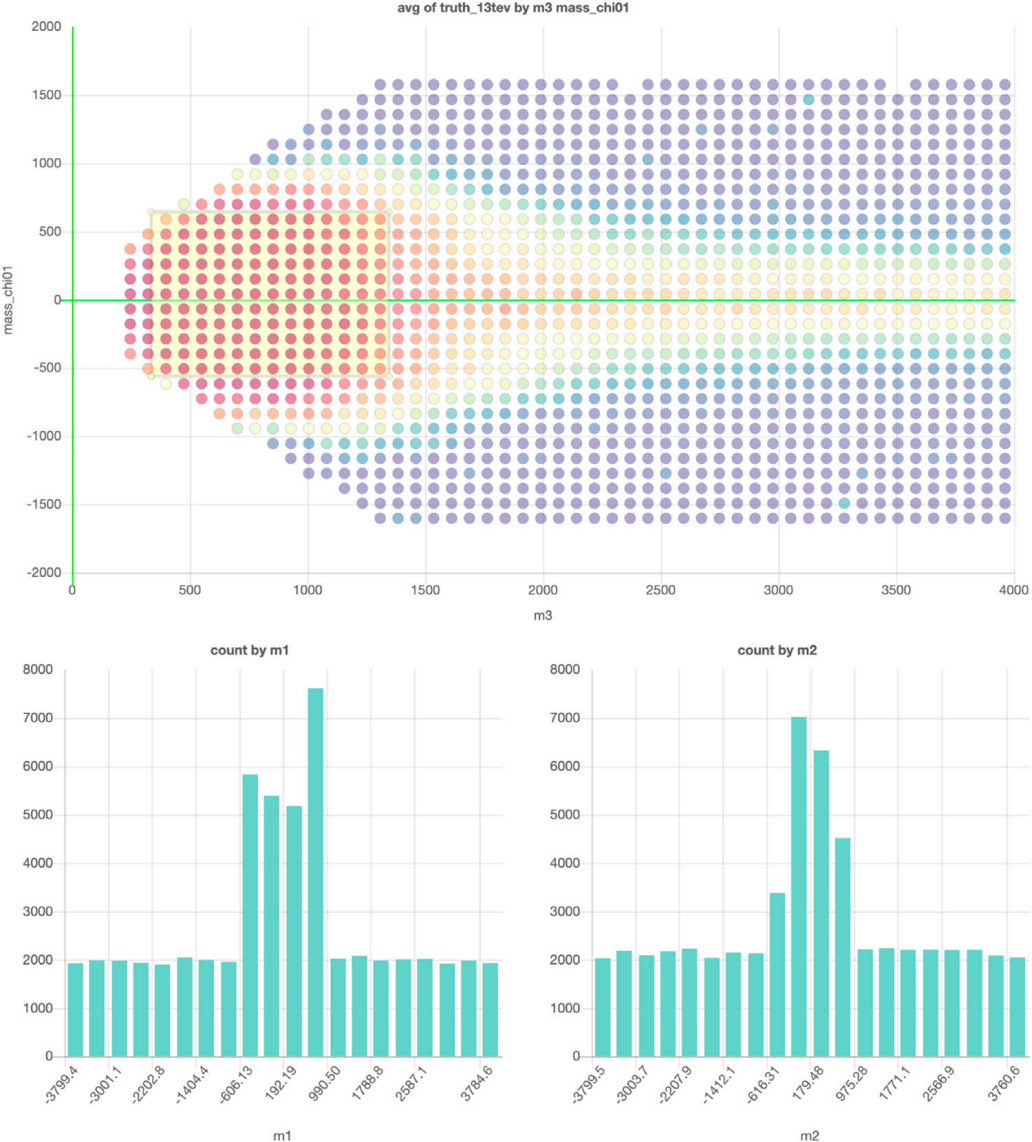
The example projection made of a subset of the data in ATLAS collaboration (2015). The graph on top shows the projection on two of the 19 free parameters and the colors indicate the average exclusion for the model configurations in each for the bins. The two graphs on the bottom can be used to make cuts on two of the remaining 17 parameters. Interactive dashboard of these graphs can be accessed here.

However, storing the data in a database with an interactive plot tool would solve this problem. The reader can then simply take the actions that interest them. In this example, a subset of the data from ATLAS collaboration. (2015) is saved so that every projection and every slice can now be plotted. Figure 4 shows an example of an exclusion plot that can be made, in which colors indicate the average exclusion in each bin. Using the dynamic links between the plots in this figure, one can make on-the-fly slices in the project plot using the plots on the bottom, allowing for a quick and full exploration of the high-dimensional dataset. This shows that a tool like this could be used to accompany papers with an effectively unlimited number of (exclusion) plots and projections based on the data used in writing the paper.

### 4.2 Example 2: Galactic Center Excess Model Solutions

In Achterberg et al. (2017) and Achterberg et al. (2015), a 19-dimensional theoretical model^1^ was tested against a measurement of an excess in the Galactic Center (GC), which was observed during the analysis of gamma rays.

In this work, a very small area of the parameter space was found that could explain this excess and lead to a particular candidate for dark matter. In these papers, various scatter plots are shown to highlight where in the parameter space these solutions are. However, these various two-dimensional plots are merely slices in the 19-dimensional parameter space. From a theoretical standpoint, we know that this parameter space is very complex: it contains delta peaks, step functions, and high-dimensional correlations. The 2D diagrams therefore do not show the complete information contained in 19D space. In addition, mapping all the combinations of 19D space would require 171 scatter plots, and this still does not show the correlations shown in more than two dimensions.

Another researcher might be interested in a scatter plot or histogram that is not in the paper because this person needs it to design an experiment to verify if the dark matter candidate exists. As this plot is not in the paper, this person needs to contact the authors and hope that they still have the results of this paper in high-dimensional format.

**FIGURE 5:**
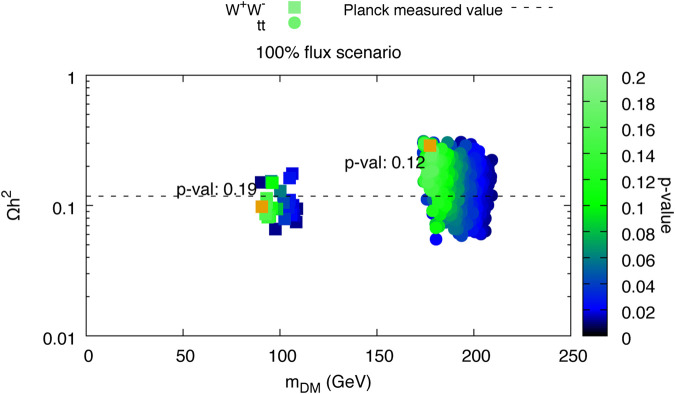
From left the right, top to bottom, plots 6c, 7c, 7b, 8 **(top left and bottom left)**, 9 **(top left and center left)**, and 6a from Achterberg et al. (2017) are visualized using SPOT. The interactive dashboard of this data set can be accessed here.

**FIGURE 6:**
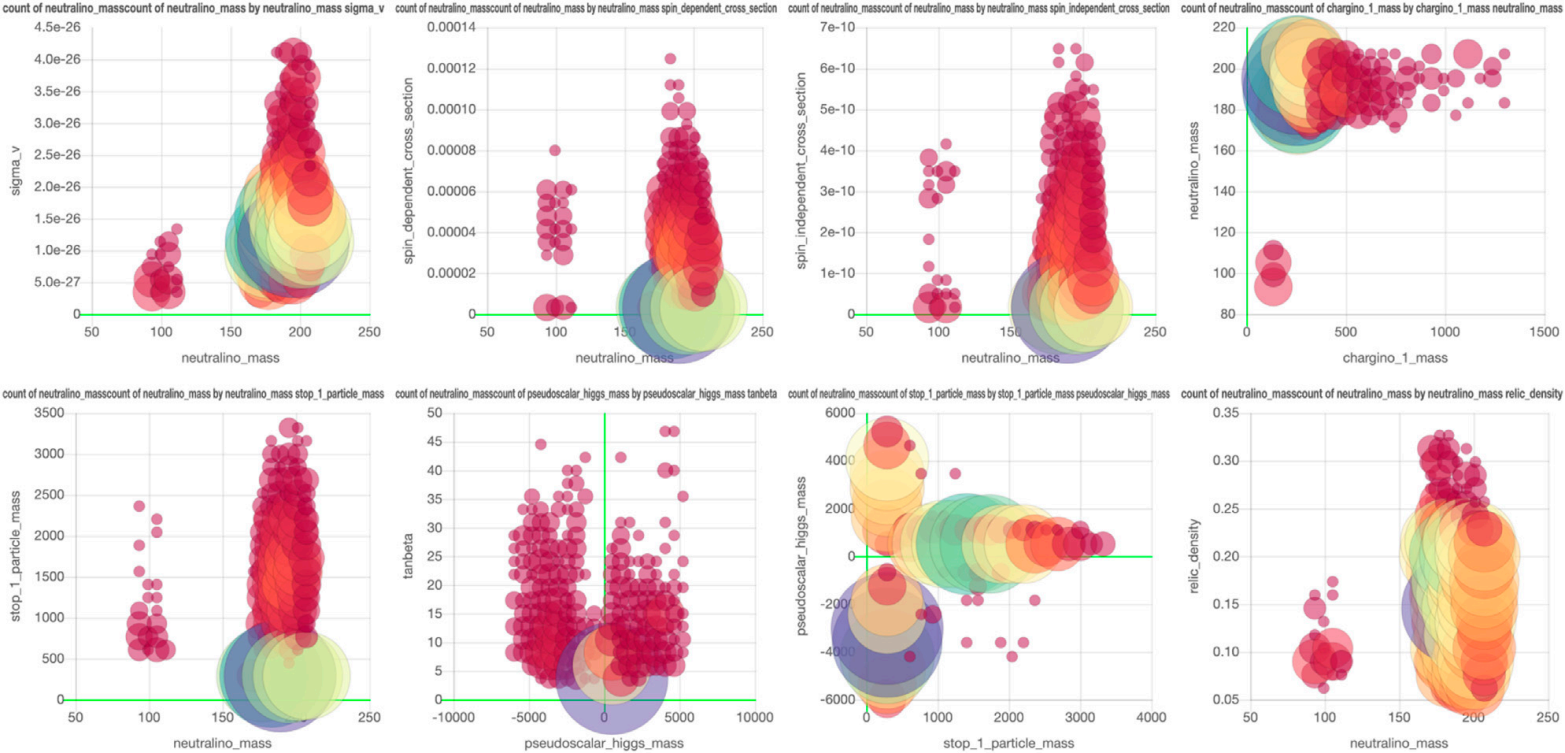
Copy of Figure 5A in Achterberg et al. (2017). This plot has been recreated in SPOT and a screenshot is shown in Figure 5. The figure is the same as the plot in the top left.

Additionally, while different theoretical models will have different input parameters, the output parameters will most likely overlap. For example, all dark matter models must predict the mass and cross section of the dark matter candidate, and all models that match the GC excess derive a likelihood. By focusing on these overlapping parameters, one can compare the results of different theoretical models in the same plots and have a very fast and convenient way to compare the results of different papers. An example of the recreation of the plots in Achterberg et al. (2017) can be found in Figures 5, 6.

### 4.3 Example 3: LHC Collider Simulation Events and Human Finding Signals

A benchmark dataset containing $>10^{8}$ simulated high-energy collision data has been provided by participants of the initiative and the 2019 Les Houches workshop (Brooijmans et al., 2020). The generated LHC events correspond to a center-of-mass energy of 13 TeV. Events for the background and signal processes are generated using the event generator MG5_aMC@NLO v6.3.2 (Madgraph) and versions above (Alwall et al., 2014). Also, a quick detector simulation with Delphes3 (cf. de Favereau et al., 2014) was performed and the high-level objects like jets, b-jets, electrons, muons, and photons have been reconstructed. The charge, object type, and four vectors (energy *E*, transverse momentum $p_{T}$, pseudorapidity η, and azimuthal angle ϕ) are stored for each object. A description of the requirements and the data structure can be found in Brooijmans et al. (2020).

This dataset can be useful for various phenomenological studies. One of the goals is to develop and compare new strategies for searching for signals from new physics. Here, it is interesting to see which regions of phase space are selected by new signal detection algorithms. Cuts of such machine learning-based algorithms are typically represented by a complicated multidimensional hypersurface of the four vectors and objects. An online interactive multidimensional visualization tool such as SPOT would allow us to quickly compare, e.g., where improvements in the algorithm should have been made.

**FIGURE 7:**
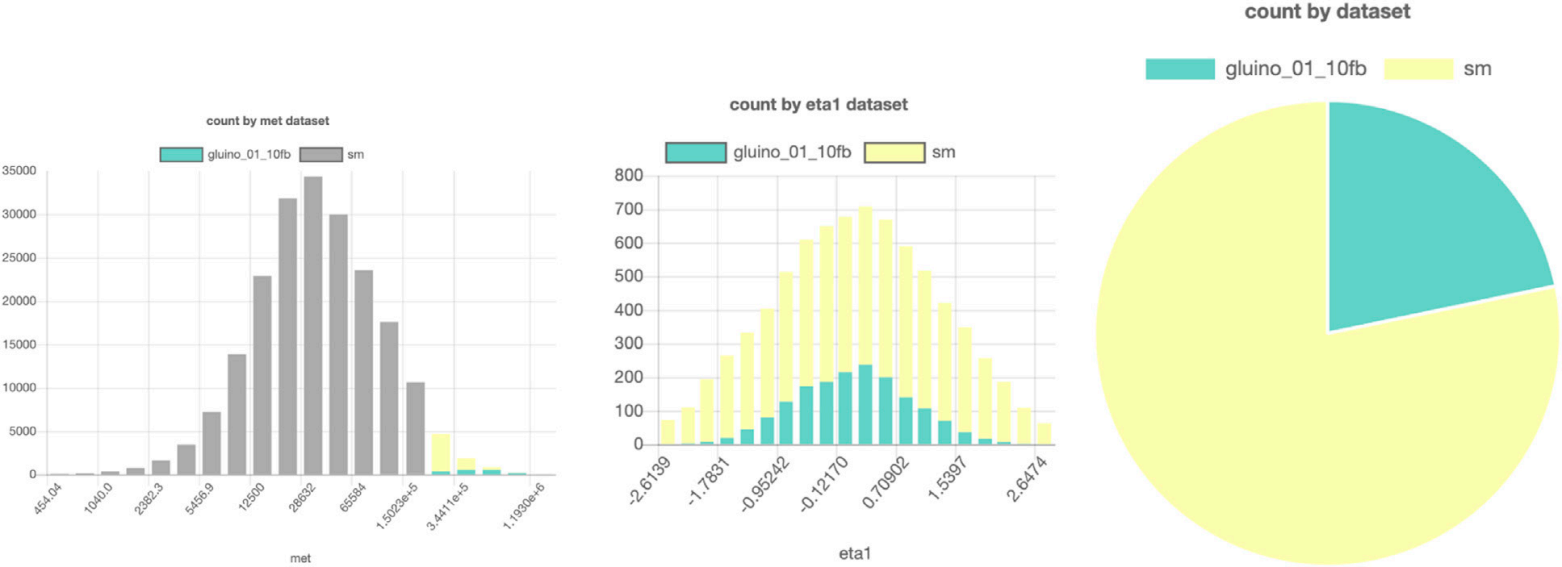
SPOT-based comparison of a possible LHC signal (here, a gluino production from supersymmetry) and background. Parts of a figure can be turned off to filter them out in the other figures. In this example, the gray bars in the left histogram are turned off, so in the middle and right figures, only the data points with high MET are shown. The interactive dashboard of this dataset can be accessed here.

An example of a SPOT session with these data can be found in Figure 7. Here, a comparison is made between a possible gluino signal from supersymmetry and the expected background events. All events are from this simulated dataset, but only 824.556 are loaded in the database to prevent high strain and necessary disk space on the server. The events are chosen randomly from the $10^{8}$ events in the full dataset.

It would be interesting to create a challenge where people can search for the signal by applying selection criteria by hand. Would they be better than machine learning-based anomaly detection algorithms? A signal region found by a “human” could be used as a “data derived” signal region in an independent dataset, similar to that proposed by ATLAS in Aaboud. (2019).

### 4.4 Example 4: Fermi Point Source Catalog

The Fermi FL8Y Point Source catalog (Fermi, 2018) contains a list of found point sources using eight years of Fermi data. It is a big table containing the locations and properties of various types of point sources. If one would be interested to quickly check where the most unresolved point sources are, the catalog has to be downloaded and then you have to write a visualization script to filter out the unresolved point sources and plot the latitude and longitude of the corresponding rows. This requires technical knowledge and is quite time-consuming for such a simple check.

**FIGURE 8:**
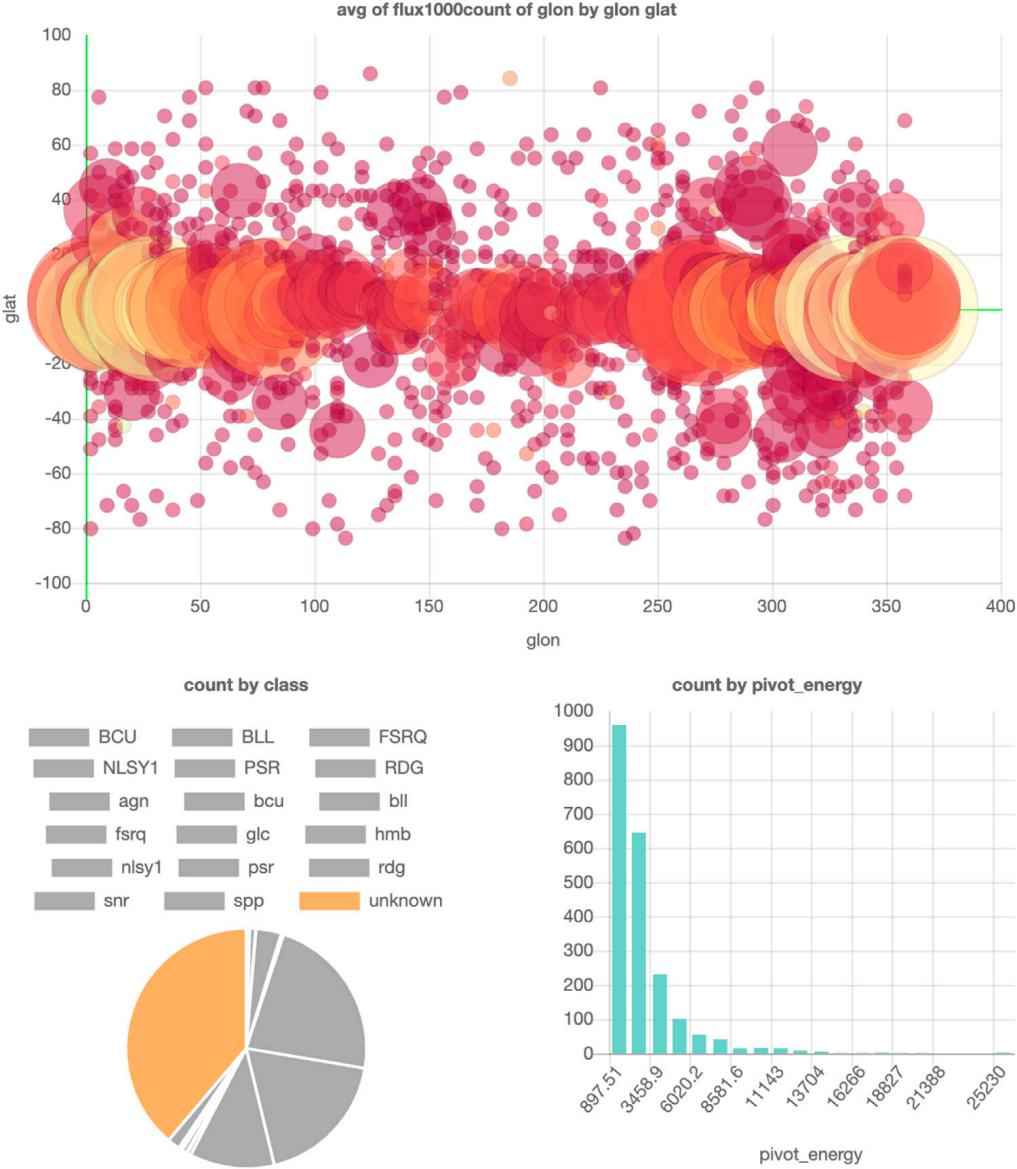
SPOT visualization of the FL8Y Fermi Point Source catalog (Fermi, 2018). Parts of a figure can be turned off to filter them out in the other figures. In this example, all point source classes except the unknown class are turned off, so the locations and pivot energies of only the unknown point sources are shown in the top and bottom right plots. The interactive dashboard of this dataset can be accessed here.

Alternatively, in SPOT, it requires only a few clicks to generate these plots and conclude that they mainly lie in the Galactic Plane (where also most of the diffuse background radiation is). An example visualization of this dataset can be found in Figure 8.

## 5 Conclusion

In this note, we propose the expansion of scientific repositories such as Zenodo to allow easy web-based visualization of data. We show some examples where such visualization could speed up science. Additionally, it would be an important step to encourage the HEP community to study physical models and publish results in their full dimensionality. This would allow and encourage a revision of the results with different model parameters, the search for anomalies (or errors) in the published data, the generalization of the results with ML, and a better comparison of the scientific publication.

We would like to emphasize that the development and the maintenance of such a tool must be a collaborative effort. We hope that the community will realize the importance of our solution so that we can build these tools together.

## Data Availability

The provenance files used in this study can be found at https://doi.org/10.5281/zenodo.4247861. Example in Section 4.1 uses a subset of the data from http://hepdata.cedar.ac.uk/view/ins1389857. The dataset for example in Section 4.2 can be found at https://doi.org/10.5281/zenodo.4255797. The dataset for example in Section 4.3 can be found at https://doi.org/10.5281/zenodo.3663386. The dataset for 4.4 can be found at https://fermi.gsfc.nasa.gov/ssc/data/access/lat/fl8y/.

## Footnotes

1 A version of the pMSSM model was also used in the previous example (Section 4.1).
